# The evaluation of right and left ventricular morphology by CMR with comparison to recipient heart after heart transplant: a surgical perspective

**DOI:** 10.1186/1532-429X-13-S1-O16

**Published:** 2011-02-02

**Authors:** Nicholas Farber, Mark Doyle, Ronald B Williams, Srinivas Murali, Peter Olson, June A Yamrozik, Robert WW Biederman

**Affiliations:** 1 Allegheny General Hospital, Pittsburgh, PA, USA

## Introduction

CMR is considered the ‘gold standard’ for non-invasive LV and RV mass quantitation. To our knowledge, this information is soley based on animal and phantom data never having been prospectively or retrospectively validated in humans, undermining its credibility. This issue is particularly important for the RV having complex geometry ill suited for mathematical modeling, placing increased importance on accurate mass quantitation. The surest way to validate the accuracy and thus the *true* gold standard of CMR derived mass is through autopsy but obviously is not feasible

## Purpose

To establish a correlation between CMR derived LV and RV mass vs autopsy mass of *ex vivo* hearts from transplants patients.

## Methods

Over a 2 year period, 21 explanted cardiomyopathic hearts were obtained immediately upon orthotopic heart transplantation from the OR. They were quickly cleaned and suspended in a saline-filled container and scanned via SSFP-SA slices defining CMR LV mass (g) (GE 1.5 T, WI). The explanted hearts were then dissected shaving the atria off at the AV valve plane with ventricles surgically separated at the interventricular septum. The weight of the LV and RV was measured via high-fidelity scale for comparison with weighed mass of 17/21 hearts suitable for study.

## Results

The CMR measured LV mass (310±74.8g) significantly predicted the actual weighed LV mass (325±85g). The Pearson product moment (PPM) correlation was 0.95 (p<0.001). The CMR measured RV mass (178±76g) significantly predicted the actual RV mass (143±63g). The PPM correlation was 0.96 (p<0.001). The CMR measured LV + RV mass (495±131) significantly predicted actual LV + RV mass (467±27g). The PPM correlation for this sample was 0.97 (p<0.001). The equation y = 1.01x - 6.6b regressed the LV (r = 0.95).

## Conclusions

CMR accurately determines LV and RV masses as compared to weighed explanted hearts, despite variable surgical removal of instrumentation (LVAD/RVAD, AICD's and often apical core removals) in the majority of hearts and the complexities of the RV in all. To our knowledge, albeit a small sample size, this represents a 'first' human CMR vs. autopsy comparison, similar to the intrepid days of initial validations of echocardiography vs. autopsy mass report by Reichek and Devereux in 1976.

